# Supplementary material for: Surface Functionalization of Bioactive Hybrid Adsorbents for Enhanced Adsorption of Organic Dyes
Source: Int J Environ Res Public Health. 2023 May 8;20(9):5750. doi: 10.3390/ijerph20095750 (PMC10177766; doi:10.3390/ijerph20095750)
Supplement: Supplementary file 1 [file ijerph-20-05750-s001.zip › ijerph-2351079-supplementary.pdf]

Supplementary Data

Surface Functionalization of Bioactive Hybrid Adsorbents for Enhanced Adsorption of Organic Dyes

Yasser M. Riyad [1,*], Taha M. Elmorsi [2], Mohd Gulfam Alam [1] and Bernd Abel [3]

[1] Department of Chemistry, Faculty of Science, Islamic University of Madinah, Madinah 42351, Saudi Arabia
[2] Department of Chemistry, Faculty of Science, Al-Azhar University, Cairo 11884, Egypt
[3] Institute of Chemical Technology, Leipzig University, Linne´-Strasse 3, 04103 Leipzig, Germany
* Correspondence: yasser.riyad@iu.edu.sa

Kinetics of MB/CV binary systems using both nonlinear and linear models of pseudo-first-order, pseudo-second-order and Elovich equation

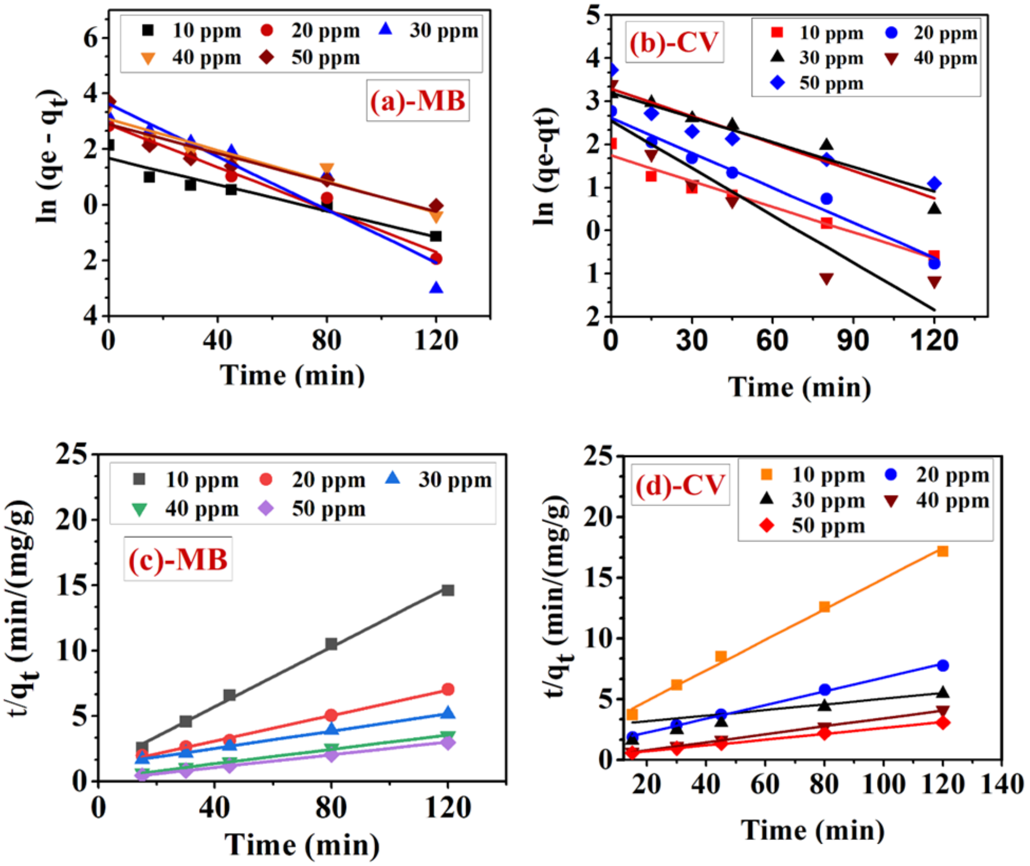

Figure S1. The linear curves for the adsorption kinetics of PSO for (a) MB in MB/CV, (b) CV in MB/CV and PSO for (c) MB in MB/CV and (d) CV in MB/CVbinary system using Cu-doped ZnO/Ext.

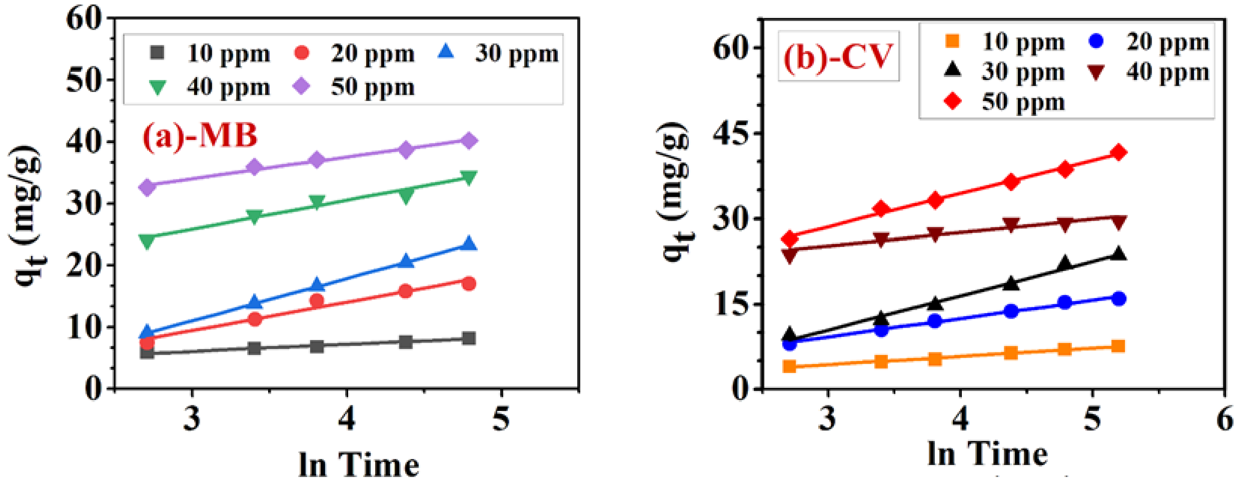

Figure S2. The linear curves of Elovich model for the adsorption of (a) MB in MB/CV and (b) CV in MB/CV binary system using Cu-doped ZnO/Ext

Table S1. Differential, nonlinear and linear forms of kinetic models used in this study

Kinetic model	Differential form	Nonlinear form	Linear form
Pseudo first-order	$\frac{dq_t}{d_t} = k_2(q_e - q_t)$	$q_t = q_e(1 - e^{-kt})$	$\ln(q_e - q_t) = \ln q_e - k_1 t$
Pseudo second-order	$\frac{dq_t}{d_t} = k_2(q_e - q_t)^2$	$q_t = \frac{k_2 q_e^2 t}{1 + k_2 q_e t}$ (7)	$\frac{t}{q_t} = \frac{1}{k_2 q_e^2} + (\frac{1}{q_e})\, t$ (8)
Elovich equation	$\frac{dq_t}{d_t} = \alpha e^{-\beta q_t}$	$q_t = \frac{1}{\beta}\ln(1 + \alpha\beta t)$	$q_t = \frac{1}{\beta}\ln(\alpha\beta) + \frac{1}{\beta}\ln t$

Note: t (min) is the time, k_1 (1/min) and k_2 (g/mg.min) are the adsorption rate constants of first- and second-ordermodels, respectively, $q_t$and q_e expressed the amount of dye adsorbed (mg/g) at any time (t) and atequilibrium, respectively, α *and* $\beta$are the initial adsorption rate (mg/g min) and desorption constant (g/mg) during each experiment respectively.

Table S2. Linear parameters of pseudo first-order and pseudo second-orderfor the adsorption of MB dye in MB/CV binary system onto Cu-doped ZnO/Ext at pH 7.5 and at room temperature.

**Linear Pseudo first-order**						**Linear Pseudo second-order**			
**Model Equation**									
[MB] ppm	$q_{e,exp}$ (mg/g)	$q_{e,cal}$ (mg/g)	k_2 (min^{-1})	R^2	**RSS**	**qe.(cal) (mg/g)**	k_1 (min^{-1})	$\mathbf{R^2}$	**RSS**
10	8.8	**5.33**	0.024	0.929	0.426	9.03	0.009	0.998	0.403
20	18.64	17.87	0.038	0.989	0.257	19.36	0.003	0.997	0.160
30	27.19	37.77	0.048	0.886	2.89	27.72	0.001	0.996	0.089
40	35.1	21.91	0.028	0.912	0.709	36.76	0.003	0.998	0.018
50	41.15	18.17	0.026	0.855	1.155	42.23	0.004	1.000	0.003

Table S3. Linear parameters of pseudo first-order and pseudo second-orderfor the adsorption of CV dye in MB/CV binary system onto Cu-doped ZnO/Ext at pH 7.5 and at room temperature.

**Linear Pseudo first-order**						**Linear Pseudo second-order**			
**Model Equation**									
[MB] ppm	$q_{e,exp}$ (mg/g)	$q_{e,cal}$ (mg/g)	k_2 (min^{-1})	R^2	**RSS**	**qe.(cal) (mg/g)**	k_1 (min^{-1})	$\mathbf{R^2}$	**RSS**
10	7.55	5.69	0.0198	0.965	0.142	8.33	0.006	0.996	1.044
20	15.88	13.60	0.0267	0.978	0.165	17.54	0.003	0.999	0.0625
30	23.67	22.22	0.0207	0.978	0.095	28.57	0.001	0.994	0.157
40	29.55	12.69	0.0365	0.874	1.908	30.30	0.008	1.000	0.002
50	41.65	24.33	0.0191	0.877	0.506	43.48	0.002	0.998	0.024
